# Suppurating Veneral Bubo; with Remarks on a Method of Subcutaneous Treatment and Report of Cases

**Published:** 1886-10-20

**Authors:** Scott Helm


### SUPPURATING VENEREAL BUBO; WITH REMARKS ON A METHOD OF SUBCUTANEOUS TREATMENT AND REPORT OF CASES.

By SCOTT HELM, M. D.,

[Read before the Chicago Medical Society, August 2, 1886.]

The object of the present communication is not so much to treat of the pathology and symptoms of suppurating inguinal bubo, as to call attention to a method of treatment I have employed in some twenty-two cases which have occurred in my own practice during the past three and a half years. I have had no hospital or dispensary facilities since I have employed this method.

The term bubo, although used in reference to an adenitis in any locality, should only be used to designate a circumscribed inflammation of the lymphatic glands of the groin.

In reference to the etiology of bubo I will enumerate chancre, chancroid and gonnorrhœa, traumatism, septicæmia, or erysipelas consequent on injury to the inferior extremity. Of these, those incident to chancroid are the most commonly met with. The bubo resulting from gonorrhœa is a very rare form of the disease. In the *resume* of cases given below there is one of which there was no doubt as to the gonorrhœal origin. The pathology of bubo, according to Armstrong, is an absorption by the lymphatic vessels of an irritating substance carried to the gland, the usual process of inflammation ensuing in consequence thereof. Sometimes this is limited to the gland; occasionally the adjacent tissues are involved. Microscopical section of such a gland shows hyperplastic formation of the cellular substance and reticulum. Rokitansky considers that the former process is more extensive than the latter.

Fox says that whenever a bubo is produced by the chancroidal pus which has found an entrance into and through the lymphatic vessels, this is certain to suppurate and to produce a chancroidal ulceration in the groin. This is not correct, for the inflammation may subside and the enlarged gland become smaller by the absorption of the neoplastic tissue. The inflammation of many of these glands subsides spontaneously, and still more so under appropriate treatment. However, there may be but little absorption and chronic induration remains, or necrosis of the gland substance results in the formation of pus. With the non-suppurating buboes this paper has nothing to do, so that I will proceed without further discussion to the description of the mode of procedure which I have adopted in those cases which go on to suppuration, and a report of its use in my hands, to which method I desire to call your attention.

The advantages which are claimed for this method of treatment are: First, that when there are two or more suppurating buboes in the same chain of lymphatics, the second or third appears higher up or further away from the initial sore than the first one. Therefore, by placing the first glandular abscess in a perfectly aseptic condition and absolutely protected from any foreign or septic matter in the external atmosphere, the encroachment of the inflammation on the neighboring glands is thereby prevented. Second, There is absolutely no scar or unpleasant after-appearance.

My attention was directed to this course in the following manner:

In April, 1884, I was consulted by a young man with an inguinal bubo on the right side, subsequent to chancroid of the frenum of the prepuce. It was already distented with pus, which was evacuated in the usual manner, and he was directed to return on the following day, which he failed to do. He did return, however, a week later, when he had another abscess in an adjacent gland. This was followed by a third, and then the three communicated by sinuses. He finally recovered, but was left with a chain of cicatrices in the right groin, which was anything but a thing of beauty. In August of this same year this young man's brother came to me with a bubo in the left groin and requested, if possible, to be saved from such a condition as his brother presented. I determined to aspirate this one and proceeded as follows: the parts were thoroughly cleansed and the needle of a hypodermic syringe introduced and one-half drachm of pus withdrawn. Then the cavity was washed out with a 2 per cent. solution of carbolic acid, and subsequently injected with 10 grains of iodoform suspended in ½ drachm of glycerine. The needle was then withdrawn, and after the parts had been wiped perfectly dry, the point of puncture was coated with a film of collodion, a compress and bandage being applied over all. He was directed to return on the following day, when no apparent change had taken place; but on the third day the swelling began to subside, and nine days after was almost wholly gone. I saw him again in October, when he reported, that there was no evidence of there ever having been any trouble. During the operation, especially during the introduction of the needle and the washing out of the cavity, he complained bitterly of the pain, and I determined at the next opportunity to employ the ether spray before beginning operations.

### CASE II.

Contracted gonorrhœa three weeks previously, which was followed by an enlargement in the left groin two weeks later. A large bubo presented itself, in which slight fluctuation was detected. This case was aspirated in the same manner as the previous case, with the exception that the ether spray was first employed for the purpose of local anæsthesia. This proceeding succeeded admirably until the washing out of the cavity, when he complained of considerable pain. The undoubted gonorrhœal origin of this case made it of especial interest. This case made an equally good recovery with the first. The greatest difficulty experienced was the inadequacy of the instrument employed, and the glycerine proved a poor menstruum in which to hold the iodoform in suspension. Soon after that I began experiments with oleic acid as a menstruum. This substance, as described in the U. S. Pharmacopœia of 1880, is a yellowish, oily liquid, gradually becoming brown, rancid and acid on exposure to the air; odorless, or nearly so; tasteless, and, when pure, of a neutral reaction. Specific gravity, 0.800 to 0.810.

Oleic acid is insoluble in water, but completely so in alcohol, chloroform, benzol, benzin, oil of turpentine, and the fixed oils. At 14° C. (57° F.) it becomes semi-solid, and remains so until cooled to 4° C. (39.4° F.), at which temperature it becomes a whitish mass of crystals. At a gentle heat the acid is completely saponified by potassium carbonate. If the resulting soap be dissolved in water and exactly neutralized with acetic acid, the liquid will form a white precipitate with test solution of acetate of lead. This precipitate, after being twice washed in boiling water, should be almost entirely soluble in ether.

Oleic acid, $C_{18} H_{34} O_2$, does not belong to the "fatty acid" series, but differs from the corresponding acid of that series (stearic acid $C_{18} H_{36} O_2$), by having two atoms of hydrogen less. It belongs to a series derived by oxidation from alcohols, which, like allyl alcohol, $C_3 H_7 OH$, have two atoms of hydrogen less than the normal mon-atomic alcohols, like propyl alcohol, $C_3 H_7 OH$. The alcohol, however, from which oleic acid is derivable is not known. Oleic acid is mono-basic, as is shown in the formula $HC_{18} H_{33} O_2$. Commercial oleic acid is obtained as a by-product in the manufacture of glycerine and candles.

It is very necessary that the preparation should be absolutely pure, and I have experienced a good deal of difficulty in obtaining such a specimen; all that I have been able to obtain in the market being dark brown and rancid. Parke, Davis & Co., of Detroit, however, made for me two specimens, one from soap and the other from oil of sweet almonds, which were what they should be.

Until quite recently I have used iodoform suspended in the oleic acid, which was a much more stable preparation than with the glycerine. Lately I have substituted iodol for iodoform, and found that it will remain in suspension in the oleic acid, and further than that, causes no symptoms of intoxication such as I experienced in two of my cases. The instrument which is now used consists of a barrel with a capacity of two drachms, and which is mounted on either side by two rings, for the fore and middle fingers, and a ring in the end of the piston for the thumb. There are three needles of different sizes, and one a canula mounted with a trochar. To these (which one may be in use) is attached the section in the center of which is a stop-cock, the opposite extremity of which is attached to the barrel by a smooth joint.

To recapitulate, the method is as follows:

The parts are thoroughly cleansed; the ether spray is used for the purpose of local anæsthesia; then the canula, attached to the stop-cock section and mounted on the trochar, is inserted into the center of the abscess; the trochar is withdrawn and the stop-cock turned in order to prevent the introduction of air. The barrel is then attached at its sliding joint and the pus withdrawn and the quantity noted. The barrel is then carefully washed and a 2 per cent. solution of carbolic acid injected and the cavity thus washed out. After this has been withdrawn the iodol or iodoform suspended in the oleic acid, is injected in like quantity to the amount of pus evacuated, after which the needle is withdrawn and the point of entrance, after being carefully dried, is coated with a film of collodion and a compress and bandage applied over all.

I have now employed this plan in twenty-two cases, and have had so far but one failure. In this case, the nineteenth, the patient went on a protracted spree the next day, and when I saw him next he had a chancroidal ulcer in the groin.

In one case—a mounted policeman—an induration like a large buck-shot still remains. This man kept right on duty during treatment. One case had a double inguinal bubo, but in no case were there two in the same chain of glands.—*Chicago Medical Journal.*

